# Epidemiological and histopathological patterns of salivary gland tumors in Cameroon

**DOI:** 10.11604/pamj.2016.23.66.5105

**Published:** 2016-03-03

**Authors:** Zacharie Sando, Jean Valentin Fokouo, Arlette Onomo Mebada, François Djomou, Alexis NDjolo, Jean Louis Essame Oyono

**Affiliations:** 1 Faculty of Medicine and Biomedical Sciences (FMBS) of the University of Yaoundé I, Yaoundé, Cameroon; 2 Pathology Service of Gyneco-Obstetric and Pediatric Hospital, Yaoundé, Cameroon; 3 Otolaryngology Service of University Hospital Center, Yaoundé, Cameroon; 4 Centre Pasteur du Cameroun, Yaoundé, Cameroon

**Keywords:** Salivary gland tumors, pleomorphic adenoma, cystic adenoid carcinoma, Cameroon

## Abstract

**Introduction:**

Tumors of salivary glands are rare. According to Johns and Goldsmith in 1989, their annual incidence is less than 1/100000 without noteworthy geographical gap. But other authors suggest that their distribution may vary according to the race and geographical location. In Cameroon, existing studies give incomplete data. Hence, we underwent this study in order to draw the general profile of salivary gland tumors in Cameroon.

**Methods:**

A retrospective study was carried out on the period spanning from January 2000 to December 2010 (11 years). It was done in nine Pathology services of different hospitals in Yaoundé, Douala and Bamenda. We consulted the archive registers of those services, retaining any patient with salivary gland tumor, whatever the histological type or location. Information gathered was the year of diagnosis, the service, the age and sex, the site of the tumor (gland) the histological type and the benign/ malignant character.

**Results:**

We recruited a total of 275 files. Women were 56% (154/275) and men 44% (121/275) of the sample. Fifty eight tumors were malignant (21.9%) while 217 were benign (78.1%). The overall mean age was 37.44 years, with extremes between 1 and 84 years. Pleomorphic adenoma (60.36%) was the most common benign tumor. Adenoid cystic carcinoma (31%), mucoepidermoid carcinoma (22.4%) and adenocarcinoma (19%) were the most common malignant tumors. Palate (66.7%), cheek (30%) and lips (3.3%) were the sites were the minor salivary glands were mostly involved.

**Conclusion:**

The differences with western world authors suggest a geographical variability of salivary gland tumors.

## Introduction

The tumors of salivary glands are rare [1–3]. According to Johns and Goldsmith in 1989, their annual incidence is less than 1/100000 inhabitants, without noticeable geographical gap andthey represent less than 5% of head and neck tumors [1]. It is postulated in the western world literature that their distribution is about 100:10:10:1 for parotid gland, sub maxillary gland, accessory salivary glands and sublingual gland respectively [2]. But recent African studies found a distribution of 1:1:1 for parotid, sub maxillary and accessory salivary glands respectively [3]. In Cameroon, existing data are partial because they are gland specific or on limited study samples. We therefore undertook this study in order to study the main histopathological and epidemiological features of the tumors of salivary glands in Cameroon.

## Methods

We carried out a retrospective, descriptive and multicentric study on the 11-year period spanning from January 2000 to December 2010. The protocol of the study received the approval of the Ethics Committee of the Faculty of Medicine and Biomedical Sciences of the University of Yaoundé I. It was conducted in nine Pathology services of Yaoundé, Douala and Bamenda. We chose those centers because together they process almost all the pathological samples of the country, so as to have the most representative national scale picture possible. The centers based in Yaoundé were the Yaoundé Gyneco-Obstetric and Pediatric Hospital, the Association Pathologie et dévelopement, the Centre Pasteur du Cameroun, the University Hospital Center, the Yaoundé Central Hospital and the Yaoundé General Hospital. In Douala were involved the Laquintinie Hospital and the General Hospital. In Bamenda the Mezam Polyclinic was selected. We used the patients’ records of these services, recruiting the file of any patient with a salivary gland tumor, either from principal or accessory salivary gland, benign or malignant. The data collected were the year of diagnosis, the service, the age, the sex, the location of the tumor (gland) and the histological type. All the pathologists from these centers had prepared their slides with the Hematein-Eosin stain. The tumors were classified using the WHO classification of 2005 [4]. Data were collected on fore-established sheets and the analysis was done with the software Sphinx Millenium 4 and Microsoft Excel 2007.

## Results

**Figure 1:**
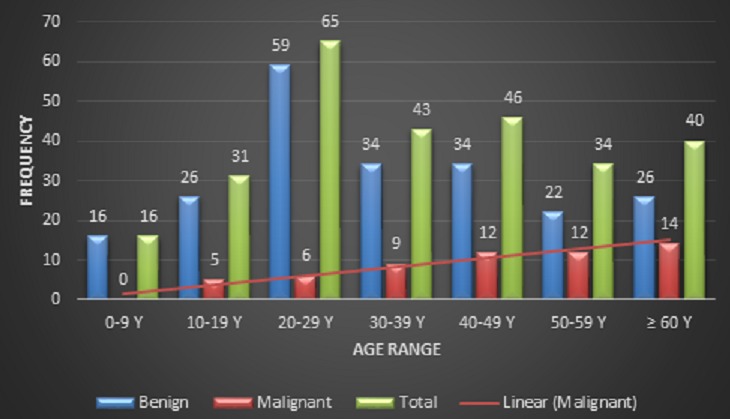
Distribution of patients according to age range and histology; the red line represents the slope of the frequency of malignant tumors

We recruited a total of 275 files from the nine hospital centers. Females were 154 (56%) and males 121 (44%) of the sample. There was an increase of the number of cases per year as from 2007, with a peak in 2009 being 45 cases. The distribution of cases per center was as follows: Mezam Polyclinic (63), Centre Pasteur du Cameroun (58), Douala General Hospital (39), Yaoundé University Hospital Center (33), Association Pathologie et Dévelopement (29), Yaoundé General Hospital (19), Douala Laquintinie Hospital (15), Yaoundé central Hospital (12) and Yaoundé Gyneco-Obstetric and Pediatric Hospital (7). The detail of the contribution of each pathology center is given in Table 1. Fifty eight tumors were malignant (21.1%) while 217 were benign (78.9%). The mean age of the patients was 37.44 (SD: 54.7) years with extremes of 1 and 84 years. Mean age was 46.9 (SD: 43.13) years with extremes of 18 and 79 years in the patients with malignant tumors versus 34.9 (SD: 39.6) years with extremes of 1 and 84 years in those with benignant tumors (p = 0.03). The distribution of tumors according to age range and histology is given in Figure 1. We obtained the sequence of 135:109:30 for parotid, submandibular and accessory salivary glands respectively, thus giving a parotid/submandibular gland ratio of 1.23 (Table 2). Pleomorphic adenoma (60.36% of benign tumors and 47.7% of all tumors) was the most frequent benign tumor. The cystic adenoid carcinoma was the most frequent malignant tumor (31%of them), followed by the mucoepidermoid carcinoma (22.4%), and adenocarcinoma (19%). There were 30 cases (20%) of accessory salivary gland involvement, of which 20% were malignant tumors. The palate (66.7%), the cheek (30%) and the lips (3.3%) were the sites affected by accessory salivary gland tumors. There were 54 inflammatory pseudo tumors, representing 24.8% of benign tumors and 19.6% of the sample (Table 3). We compared our results to those of other authors. It shows a similarity between African results, contrary to Western World literature (Table 4).

**Table 1:** Contribution of each pathology service per year

	YCH	UHC	YGH	YGOPH	CPC	DGH	DLH	APD	MP
2000	1	0	0	0	2	1	0	0	1
2001	0	2	2	1	5	6	2	2	3
2002	2	2	1	0	1	1	0	1	2
2003	1	2	2	0	4	5	2	2	2
2004	2	3	1	1	3	1	2	1	1
2005	1	3	4	0	7	4	1	2	2
2006	1	2	2	1	5	4	1	3	4
2007	2	5	2	0	11	5	2	3	11
2008	1	2	1	1	6	3	2	3	11
2009	1	5	3	2	6	5	2	7	12
2010	0	7	1	1	6	4	1	5	13
**Total**	**12**	**33**	**19**	**7**	**58**	**39**	**15**	**29**	**63**

YCH: Yaounde Central Hospital, UHC: University Hospital Center, YGH: Yaounde General Hospital, CPC: Centre Pasteur du Cameroun, DGH: Douala General Hospital, DLH: Doula Laquinitine Hospital, APD: Association Pathologie et Dévelopement, MP: Mezam Polyclinic.

**Table 2:** Distribution of tumors according to the site

	Distribution of tumors according to site						
**MALIGNANT**		**CAC**	**MEC**	**AdenoK**	**Lymphoma**	**Others**	**Total**
	Parotid	10	8	7	0	10	35
	Submandibular	4	5	2	2	4	17
	Accessory	4	0	2	0	0	6
	Sublingual	0	0	0	0	0	0
	Total	18	13	11	2	14	**58**
**BENIGN**		**Pl. adenoma**	**Whartin's**	**Lymphangioma**	**Others**		**Total**
	Parotid	63	0	0	37		100
	Submandibular	47	2	1	42		92
	Accessory	22	0	0	2		24
	Sublingual	0	0	0	1		1
	Total	132	2	1	82		**217**

**Predominant sites were parotid and submandibular glands.**

**CAC= Cystic adenoid Carcinoma, MEC= Mucoepidermoid carcinoma, Adeno K= adenocarcinoma, Pl. adenoma= pleomorphic adenoma**

**Table 3:** Distribution of the tumors referred to as « others »

	Histology	Frequency
**Malignant**	Aciniccelladeno carcinoma	2
	Carcinoma Epidermoid carcinoma	7 5
**Benign**	Inflammatory pseudo tumor	54
	Adenoma	5
	Epidermoidcyst	2
	Sialolithiasis	11
	Oncocytoma	3
	Adenomyoepithelioma	1
	Cystadenoma	1
	Fibroustumour	1
	Mikulicz'stumor	5
Total		97

This group was dominated by benign tumours.

The inflammatory pseudo tumor represented 55.67% of them.

**Table 4:** Comparison of our results with those of other authors

		Present study (Cameroon 2013)	Ochicha *et al* Nigeria, 2009	Vuhahula *et al*. (Ouganda, 2004)	El-Gazayerli Egypt, 1964	Speight *et al*. (Review article 2002)
**Sample size**		275	78	268	78	-
**Benign tumors**		78.1%	56.4%	54%	85.9%	
**Malignant tumors**		21.9%	43.6%	46%	14.1%	
**Most frequent tumor**	**Benign**	Pl. adenoma 47.7%	Pl. adenoma 48.7%	Pl. adenoma 40.9%	Pl. adenoma 75.6%	Pl. adenoma 50%
	**Malignant**	CAC* 6.6%	MEC** 23.1%	CAC* 13.4%	Carcinoma 8.9%	MEC** 10%
**Sex predominance**		Female	Female	Female	Male	Female
**Meanage**		37. 4 yrs	36.5yrs	38.1 yrs	36.3yrs	-
**Peak incidence**	**Benign**	20-30 yrs	21-30	43,1 yrs	31-40 yrs	-
	**Malignant**	60 yrs& +	60 yrs& +	33.5 yrs	40 yrs	-
Cystadenilymphoma		0.7%	0	0	10.25%	-
Parotid/submandibular ratio		1.23	1.9	1.15	3	8 to 10

* Cysticadenoid carcinoma

** Mucoepidermoid carcinoma. Pl.=Pleomorphic.

We noticed a rarity of Wharthin's tumor in the majority of African studies as well as a very low parotid/submandibular ratio.

## Discussion

There is a boom of tumoral diseases in Africa in general and Cameroon in particular. It is estimated that 12,000 to 14,000 new cases are diagnosed each year in the Country [5, 6]. Tumours of head and neck follow the same progression. In this retrospective work on the tumors of salivary glands, we found a predominance of benign tumors (78.1%), pleiomorphic adenoma being the most frequent tumor and cystic adenoid carcinoma the most frequent malignant tumor. The parotid/submaxillary ratio of 1.23, far less than the 8 to 10 of western world literature. Accessory salivary gland involvement represented 20% of cases, of which 20% were malignant.

There was a raise of the number of tumors per year between 2000 and 2007. This was also noticed by Bahiru in Ethiopia [7]. It is due to a better access to pathology diagnosis thanks to the creation of new laboratories on one hand and to a better sensitization of the health personnel on cancer and the importance of pathological diagnosis on the other hand. The fact that Polyclinic Mezam alone has up to 22.9% of cases is because this center is almost the only situated in a highly populated zone and thus receives patients from 3 regions (North-West, West and Littoral). In the big cities Yaoundé and Douala, a greater number of laboratories allows a sharing of patients. The parotid/submandibular ratio obtained (1.23) is near to those found by other African authors [2], but far from the one found in most of western world studies (8 to 10) [8]. This supports the fact that the distribution of those tumors is not uniform and varies greatly depending on geographical localization. Besides, the parotid as the most involved site is a universal finding in the literature [8–11].

The mean age of patients was 37.4 years, similar to what Kayembe (37.4) [10] and Vuhahula (38.4) [2] found in Ouganda and Congo respectively. It was higher than the 30 years found by Bahiru [7], what he explained by the fact that in those regions persons of the third age are more attracted to alternative medicines. The age range most affected by malignant salivary gland tumors was the 60 and above (24.1%). Evesonet al found a peak of malignant tumors at 70 years [9]. The 20-30 age range was the most affected by benign tumors (27.2%). It is younger compared to the findings of Eveson [9] and Onyango [11] who found peaks of incidence at 60 and 40-59 years respectively. These differences suggest a variation of the distribution of those tumors according to the site, due to different influencing factors. Especially, the longer life expectancy, the development of means of prevention and the better quality of life in the western world [12].

Women were predominant in the sample, with a male/female sex ratio of 0.78. Kayembein Congo and Vuhahula in Ouganda found similar results with 0.8 and 0.76 respectively [2, 10]. This predominance can be due to the women's tendency to better care about their cosmetic appearance, thus seeking more often medical help when a tumor deformity appears. Malignant tumors also predominate in the women (51.7%). It was also found by Vuhahula (52.4%), unlike Ben Brahimand El-Gazayerliwho instead found the predominance in males [8, 13].

There was a predominance of benign tumors (78.1%). This was similar to the results of Ben Brahim [13] and Ouoba [14] with respectively 88.3% and 83.3% of benign tumors. Vuhahula [2] and Masanja [15] found an almost equal distribution: 54% of benign tumors and 46% of malignant tumors. This is due to the fact that in our study, the regions concerned have a good health coverage and the health personnel performs surgery straightaway in front of a clinical suspicion of benign tumor, surgery easily accepted by the patients in this context, contrary to when malignancy is suspected, then preferring traditional medicine. The benign tumors could also have been over diagnosed in our sample since some of the pathological characteristics of pleomorphic adenomas are similar to those of carcinomas. So, some pathologists here as elsewhere classify low grade malignant tumors in the benign tumors [4, 16]. In addition to that, immunohistochemical methods of pathology diagnosis are not yet used in the study centers.

Proportionally in this study, the parotid gland is less often the site of tumor (49%) than in the western studies (70%) [16], while the submandibular gland is more often, with 39% versus 10-15% [1]. This has been found by other African authors [2, 14]. Some like Aboul Nasr (1961) emitted the hypothesis that endemic parotiditis could decrease the susceptibility of parotid gland and increase that of the submandibular gland to neoplasms [8]. El-Gazayerli et al. also noticed that malnutrition altered more the serous cells than the mucous, thus decreasing the susceptibility of parotid gland to neoplasms [8].

Pleomorphic adenoma represented 47.7% of the tumors of salivary glands, being the first. This is constant finding for all the authors. Masanja and Vuhahula found similar proportions of 44.4% and 40.9% respectively [2, 15], contrary to Ben Brahim (61.7%) [13] and Bahiru (58.5%) [7]. The cystic adenoid carcinoma was the most frequent malignant (6.6%), followed by the mucoepidermoid carcinoma (4.7%). Vuhahulain Ouganda, Kayembe in Congo and Masanja in Tanzania also found cystic the same occurrence 13.4%, 15.9% and 24.8% respectively. But Ben Brahim, Bahiru Speight et Ochicha [17] found the mucoepidermoid carcinoma as the most frequent malignant tumor (3.9%, 10.8%, 10% and 5.12% respectively) ahead of cystic adenoid carcinoma (2.2%, 10.2%,4% and1.28%). Cystadenolymphoma (Whartin's tumor) represented 0.7% of the sample. Its rarity is constant in African series [3, 10], while in western countries it accounts for 5 to 20% of all tumors of salivary glands [2]. The young age of African patients has been advanced to explain the gap [8]. But this argument is not sufficient since the age range of their peak in African series contains the mean age of Western World series [2].

Twenty two per cent of the tumors of accessory salivary glands were malignant. Ben Brahim found a lower proportion of malignancy, 9.1%, contrary to western literature which reports as much as 50% of tumors of those glands being malignant [16]. We have no explanation to this difference. The limits of this study are its inability to clearly discriminate the factors influencing the distribution of salivary gland tumors, since we didn't have sufficient data to run parametric tests.

## Conclusion

This study shows a distribution of salivary gland tumors different from that of western countries and aligning with other African authors, strengthening the hypothesis that salivary gland tumor present with regional variations which could be related to endemic infections and nutritional factors amongst other things. But prospective studies need to be carried out on bigger samples in order to better discriminate the influencing factors.

### What is known about this topic?

- Salivary gland tumors are rare.
- The parotid/submandibular ration equals 8 to 10.
- 80% of the tumors arise from the parotids.

### What this study adds

- Parotid/submandibular ratio is 1.23.
- Cystadenolymphoma is very rare.
